# Match Demands of Women’s Collegiate Soccer

**DOI:** 10.3390/sports8060087

**Published:** 2020-06-12

**Authors:** Andrew R. Jagim, Jason Murphy, Alexis Q. Schaefer, Andrew T. Askow, Joel A. Luedke, Jacob L. Erickson, Margaret T. Jones

**Affiliations:** 1 Sports Medicine, Mayo Clinic Health System, Onalaska, WI 54650, USA; erickson.jacob@mayo.edu; 2 Exercise & Sport Science, University of Wisconsin—La Crosse, La Crosse, WI 54601, USA; jmurphy@uwlax.edu (J.M.); aschaefer@uwlax.edu (A.Q.S.); jluedke@uwlax.edu (J.A.L.); 3 Department of Kinesiology and Community Health, University of Illinois at Urbana-Champaign, Urbana, IL 61801, USA; askow2@illinois.edu; 4 School of Kinesiology, George Mason University, Manassas, VA 22030, USA; mjones15@gmu.edu

**Keywords:** physiological demands, athlete monitoring, training load, football

## Abstract

Research describing the match and specific positional demands during match play in women’s collegiate soccer is limited. The purpose of the study was to quantify the match demands of National Collegiate Athletic Association (NCAA) Division III soccer and assess position differences in movement kinematics, heart rate (HR), and energy expenditure. Twenty-five Division III women soccer players (height: 1.61 ± 0.3 m; body mass: 66.7 ± 7.5 kg; fat-free mass: 50.3 ± 6.5 kg; body fat%: 25.6 ± 5.1%) were equipped with a wearable global positioning system to assess the demands of 22 matches throughout a season. Players were categorized by position (goal keepers (GK), center defenders (CB), flank players (FP), forwards (F), and center midfielders (CM)). Players covered 9807 ± 2588 m and 1019 ± 552 m at high speeds (>249.6 m·m^−1^), with an overall average speed of 62.85 ± 14.7 m·m^−1^. This resulted in a mean HR of 74.2 ± 6% HR max and energy expenditure of 1259 ± 309 kcal. Significant and meaningful differences in movement kinematics were observed across position groups. CM covered the most distance resulting in the highest training load. FP covered the most distance at high speeds and mean HR values were highest in CM, CB, and FP positions.

## 1. Introduction

The use of wearable technologies, such as global positioning systems (GPS) with integrated tri-axial accelerometry, and radio-frequency local positioning systems (LPS) provides insight into the external load placed upon athletes and allows for the quantitation of specific training demands for different levels of competition. Depending on the specifications of the wearable device, physiological responses may also be recorded, thus providing the ability to quantify the internal load (i.e., heart rate) imposed on the athlete in addition to external load (i.e., distance, speed, number of sprints, etc.) [1]. This information enables practitioners to manage player load, make informed coaching decisions, help optimize recovery, direct nutritional interventions, and reduce injury risk. Previous research has indicated the existence of a relationship between training load and injury in team sport athletes, whereby excessive training load predisposes athletes to increased risk of injury [2,3]. However, the threshold for what constitutes an excessive amount depends upon many factors, including an athlete’s training history, past medical history, psychological factors, genetic factors, sport, and training period. Moreover, failure to provide an adequate stimulus throughout the preseason or preparatory period may also increase an athlete’s risk of injury due to a lack of adequate physiological preparedness and base level of conditioning, particularly in younger athletes [4]. By understanding the demands of specific sports, practitioners can develop sport-specific conditioning programs to optimize training adaptations and develop preseason practice schedules to elicit the required adaptations while minimizing the potential for overtraining or injury.

Several publications have reported the match demands of soccer. However, the majority of these data are in elite (e.g., professional/international) soccer athletes, concentrated on men’s divisions, or have focused on small-sided games. Match play in these settings varies in terms of rules, caliber of competition, and physical demands when compared to the collegiate level [5,6,7,8,9,10,11]. Generally, when compared to the elite level of the same sex, differences in match play result in shorter distances covered total, shorter distances at high speeds, and lower velocities at the collegiate level [9,10]. Notable differences in movement characteristics and training load have also been reported regarding the match demands of collegiate play compared to elite levels of competition, particularly across certain position groups [5,9,10,12]. Despite the growing popularity of women’s soccer, information in regard to the physiological demands and movement kinematics of match play at the collegiate level is limited within the literature. Further, there continues to be ongoing debates within the sporting community on the differences in the overall caliber of play between men’s and women’s leagues. Pedersen et al. [13] presented a unique comparison of men’s and women’s soccer by scaling the match demands according to anthropometric and physiological sex differences. The authors [13] concluded that differences in the style of play between sexes are likely a result of logical and strategic adaptations to account for the notable anthropometric and physiological differences between sexes. Recently, researchers quantified the match demands of National Collegiate Athletic Association (NCAA) Division I men’s and women’s soccer [5,12] and found similar total and high speed distances during match play with differences mostly limited to peak and mean velocities between sexes. Previous research at the collegiate level has also examined accumulated training loads throughout the season and reported that starters accumulated greater total distances covered, training impulses, and number of accelerations throughout the season compared to reserves [14]. Therefore, it may be important for practitioners to individualize training load prescription by starter status, sex and based on the level of competition in order to meet the demands of match play specific to the caliber of play. Since match demands have been shown to be higher at the elite and international level, it is important for collegiate athletes not to undergo workloads appropriate for players competing at the elite level, as there could be a higher risk of injury. Additionally, further research examining the differences in match play across different levels of competition is needed to better direct long-term athlete development and provide objective evidence for the management of workloads.

Rules, game flow, and tactical strategies differ across levels of the sport, likely impacting the specific demands of match play at each level of competition. For example, at the elite level, three substitutions are allowed per match with no re-entry allowed, and time is added to matches with no clock stoppages. Further, in the event of overtime, two 15 min periods are played without a “golden goal” to decide the outcome. At the NCAA level, the clock stops on goals, injuries, and cards with no time added, and re-entry is allowed. Overtime consists of two 10-minute periods, and a “golden goal” can decide the outcome. It is therefore important to continue examining the match demands of women’s soccer across different levels of play as notable differences in anthropometrics, strength, and fitness status between sexes likely hinder the ability to translate findings from men’s soccer to the women’s game. Currently, there is limited information available on the match demands of NCAA Division III women’s soccer, yet there are 446 universities with programs within the United States and a higher number of Division III women’s soccer athletes than at the Division II and I level [15]. Therefore, the purpose of this study was to quantify the match demands of NCAA Division III women collegiate soccer and examine differences by position group.

## 2. Materials and Methods

### 2.1. Study Design

**Figure 1:**
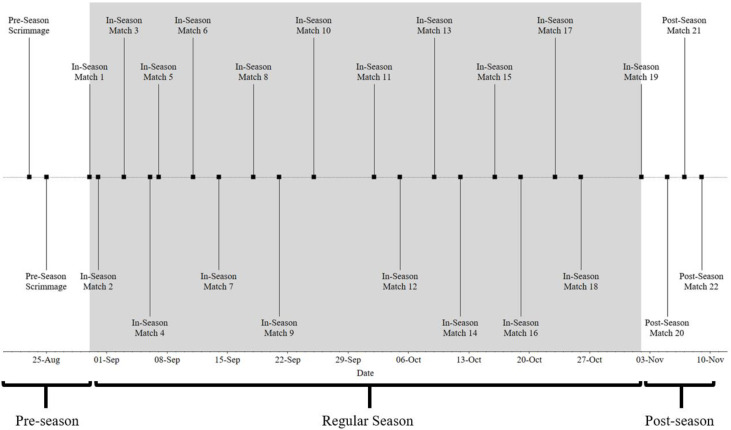
Overview of 2019 competition schedule and phases of season.

Data from 22 competitive matches collected during the 2019 competitive season were analyzed from “starters”, who were defined as those that participated in >50% of a match’s duration (Figure 1). 

This threshold was used to create a uniformity of full match demands and to allow for comparisons to be made across position groups to avoid a lowering of mean values by non-starters. A total of 241 match files were included in the final analysis, while 325 were excluded due to non-starter status. Prior to the start of the season, all players were invited to an informational meeting at which time details of the study and their participation were verbally explained. Starting during the preseason training period, all players wore the GPS-based monitoring units throughout the duration of each practice. Players were instructed to position the monitors in accordance with manufacturer guidelines once they were on the field, prior to starting the warm-up. The monitors were removed at the end of each match. Players followed the same protocol for all matches in the competitive season. To allow for comparisons in match demands across position groups, the following position group categorization was used: center defenders (CB; n = 56 files), flank players (FP; n = 64 files), forwards (F; n = 12 files) and center midfielders (CM; n = 86 files), and goal keepers (GK; n = 23 files).

### 2.2. Subjects

Twenty-five NCAA Division III women soccer players (age: 19.67 ± 1.07 years.; height: 1.61 ± 0.3 m; body mass: 66.7 ± 7.5 kg; fat-free mass: 50.3 ± 6.5 kg; body fat%: 25.6 ± 5.1%) competing for a university located in the upper Midwest region of the United States were included in this study. This study was conducted according to the Declaration of Helsinki guidelines and all procedures were approved by the University’s Institutional Review Board for use of human participants in research. Written consent was obtained from all subjects prior to data collection.

### 2.3. Anthropometrics and Body Composition

Height was assessed using a wall mounted stadiometer. Body composition was assessed using air displacement plethysmography (BOD POD, Cosmed USA Inc., Concord, CA, USA) for the determination of body mass, body fat percentage, fat mass, and fat-free mass.

### 2.4. Global Position System, Accelerometry, and Heart Rate

All players were equipped with a GPS monitoring system with an integrated accelerometer and heart rate sensor (Polar TeamPro^TM^ Polar Electro, Oy, Kempele, Finland). The validity and reliability of GPS units for characterizing movement demands during sport-related activity has been studied by a number of authors. In particular, 10 Hz GPS units, such as the one used in this study, provide a valid and reliable estimate of kinematic data with sufficient inter-unit reliability for comparisons between athletes [16]. To further promote reliability, players wore the same unit for each practice and game throughout the season. Player demographic information was entered into the manufacturer software associated with the monitoring system and used to predict aerobic capacity and max heart rate based on age and manufacturer algorithms. Max heart rates (HR) were continually adjusted throughout the preseason to provide the most accurate and up-to-date measure of maximal HR. Heart rate zones were used to quantify match intensity and defined as: Zone 1 = 50–60%, Zone 2 = 60–70%, Zone 3 = 70–80%, Zone 4 = 80–90%, Zone 5 = 90–100%. The software provided a proprietary metric referred to as Training Load, which was calculated from heart rate response and duration of activity, and it was presented in arbitrary units. At the end of each match, sensors were removed from the players, loaded to a docking station, and synced to a cloud-based software operated by the manufacturer. Then, data were exported and databased for later analysis. 

### 2.5. Movement Kinematics

The monitoring system provided a count of accelerations and decelerations using the following thresholds for categorization: Low: ±0.5–1.99 m·s^−2^, Moderate: ±2.00–2.99 m·s^−2^, High: ±3.00–50.0 m·s^−2^. The following thresholds were used for the determination of distance covered in each speed zone: Walk/Stand: <6.99 km·hr^−1^, Jog: 7.0–14.99 km·h^−1^, Run: 15.0–18.99 km·h^−1^, Sprint: >19.00 km·h^−1^. High-speed distance was calculated as a combination of run and sprint speed zones. Sprints were also counted in an accumulating fashion and were defined as any movement resulting in an acceleration >2.8 m·s^−2^.

### 2.6. Statistical Analysis

Data across all matches were collapsed and presented as mean ± SD for descriptive purposes. Differences in movement characteristics among position groups were examined using a one-way analysis of variance (ANOVA). Alpha was set at *p* ≤ 0.05 for the determination of statistical significance. Pairwise differences were used to calculate Cohen’s d effect size along with 95% confidence intervals to determine the magnitude of differences. The effect sizes were interpreted using the following criteria: 0.2 = trivial, 0.2–0.6 = small, 0.7–1.2 = moderate, 1.3–2.0 = large, and >2.0 = very large. All data were analyzed using the Statistical Package for the Social Sciences (IBM SPSS Statistics for Windows, Version 25.0: IBM Corp. Armonk, NY, USA).

## 3. Results

A summary of the movement characteristics, internal load, and energy expenditure by position group is presented in Table 1. 

**Figure 2:**
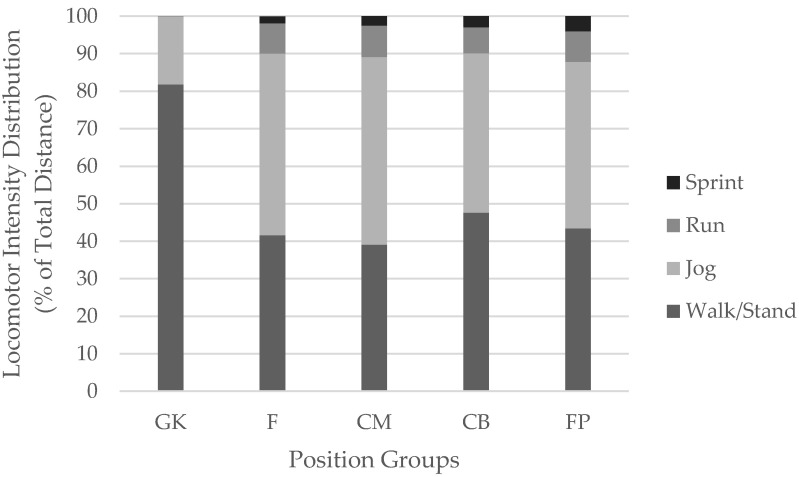
Distribution of Distance in Speed Zones. GK = goal keepers; CB = center defenders; FP = flank players; F = forwards; and CM = center midfielders. Acceleration/Deceleration thresholds: Low: ±0.5–1.99 m∙s^−2^, Moderate: ±2.00–2.99 m∙s^−2^, High: ±3.00–50.0 m∙s^−2^. The following thresholds were used for determination of speed Walk/Stand: <6.99 km·h^−1^, Jog: 7.0–14.99 km·h^−1^, Run: 15.0–18.99 km·h^−1^, Sprint: >19.00 km·h^−1^.

Players covered an average distance of 9807 ± 2588 m total and 1019 ± 552 m at high speeds, with an overall average speed of 62.85 ± 14.7 m·min^−1^. This resulted in a mean HR of 74.2 ± 6%, a calorie expenditure of 1259 ± 309 kcal, and a training load of 241 ± 75 AU per match. Significant and meaningful differences in movement kinematics and internal load were observed across position groups, as presented in Table 2. Figure 2 presents a summary of the distribution of distances covered in each speed zone by position group.

## 4. Discussion

The primary aim was to quantify the match demands of NCAA Division III women’s soccer. The development and integration of microtechnology, GPS, and wearable devices has provided novel insights into the physiological demands of soccer across different levels of play. Previous studies have examined the match demands of elite soccer. However, limited information is available in regard to collegiate women’s soccer yet close to 30,000 athletes participate at this level. In the current study, athletes covered approximately 9800 m of total distance per match and 1019 m (approximately 10%) at speeds faster than jogging (high-speed distance). The overall mean velocity of match play was 63 m·min^−1^ with an average of 15 sprints per match. This distance and speed of play elicited a heart rate response of 142 bpm or 74% of HR max with peak HR values of 197 bpm equating to 101% HR max. Overall, the match demands of NCAA Division III women’s soccer appear to be comparable with NCAA Division I play as previous research has reported an average distance of 8900–9900 m and an average high-speed distance of 1200–1900 m [5,12]. Recently, Sausaman et al. [12] used similar velocity thresholds as those in the current study (>15 km·h^−1^) and reported an average match distance of 9486 m (9186–9786 m) across 4 seasons of play in NCAA Division I women’s college soccer with a high speed distance of approximately 1442 m. These match demands are similar to those of the current study with the exception of a slightly greater amount of high-speed distance in Division I (Div. I: 1442 versus Div. III: 1019 m). In Division I men, an average HR of 77–79% HR max per match throughout a 24-match season was observed, which is slightly above the HR values observed in the current study [5]. Interestingly, although the distances covered and HR responses are comparable between Division I men and Division III women, the average velocity of Division I men match play was 87–97 m·min^−1^, which is faster than the 63 m·min^−1^ observed in the current study. Previous research has reported elite level men typically cover more distance and at higher speed thresholds than elite women [6]. However, sex differences at the collegiate level appear to exist only in mean and peak velocities of match play, which may be partially attributable to anthropometric and physiological differences between sexes [13].

In the current study, locomotor intensity/time was distributed as 46% walking/standing, 44% jogging, 7% running, and 3% sprinting, which is consistent with previous match play in collegiate males [5]. When using similar thresholds to categorize acceleration and deceleration intensity and frequency of occurrence, men’s Division I soccer appears to require more mid to high level accelerations (815 versus 74) and decelerations (660 versus 85) than the current study. Such contrasts indicate that men’s soccer may require a higher frequency of starting and stopping performed at higher velocities compared to the women’s style of play.

The overall match demands observed in the current study are slightly below those reported in elite women’s soccer matches. Mohr et al. [10] reported total match distances of approximately 10,300 m and approximately 1800–2000 m covered with high-intensity running and sprinting. Similarly, Krustrup et al. [9] noted average distances of 10,300 m (range of 9700–11,300 m) and 1300 m covered with high-intensity running in elite female soccer players yielding an average HR of 87% of HR max (167 bpm) and peak HR values of 97% of HR max. As noted by Sausaman et al. [12], the differences in match demands between the college and elite international level suggest that it may be a difficult transition from college to higher level play. It is recommended that caution be exercised when comparing movement profiles across studies as different technologies may utilize different thresholds for locomotor classification and may have varying degrees of accuracy when assessing movement. In the current study, the average number of sprints (i.e., acceleration of >2.8 m·s^−2^) was 15, which is lower than that reported in elite women (i.e., 17–21), yet previous research utilized different thresholds [11]. Therefore, it is important to note differences in software thresholds and analytical techniques when drawing comparisons within the literature, as different systems and kinematic classifications may yield varied outcomes. For example, Nakamura et al. [11] utilized two different thresholds (Fixed: >20 km·h^−1^; versus individually based threshold: >90% of 20 m maximal sprint velocity) to determine sprint activity, which resulted in different values depending upon the method.

When comparing the match demands from the current study to other field-based women sports with a similar, intermittent style of play, the distances observed in the current study are higher than those reported in collegiate women’s lacrosse [17]. Devine et al. [17] observed an average distance of 4733 m (1259–7811 m) per lacrosse game with an average of 656 m completed at high speeds, which is lower than the 9800 m of total distance and high-speed distance of 1019 m observed in the current study. International women lacrosse players reportedly cover less distance with an average of 3792 m per match and a mean heart rate of 75% of HR max [18]. Further, previous research with women’s field hockey reported average distances of 5558 m per match with 589 m completed at high speeds while eliciting a mean heart rate of 86% of HR max [19]. The increased heart rate response in field hockey may be attributed to the increased upper body involvement of the sport, higher level of contact, and shorter bursts of maximal intensity inherent to the sport.

The current study is the first to examine mean energy expenditure values per match across an entire season using an HR-derived estimate. Previous research examining energy expenditure in women soccer players reported lower values than the current study’s approximate average of 1300 kcals per match and average rate of 8.19 kcal·min^−1^ [20,21]. However, Reed et al. [21] did not provide data specific to match related energy expenditure and estimated energy expenditure using a predictive formula, while Mara et al. [20] reported data from a single match. 

A secondary aim was to examine differences in match demands between position groups. As in many sports, each position requires a unique tactical strategy and style of game play, which has been shown to result in different movement profiles and physiological demands in elite men [7,22] elite women [10] and collegiate men [5]. In the current study, the CM covered the greatest distances on average with meaningful differences and moderate effect sizes observed between CB and F. The greater distance covered by CM seemed to correspond to a greater training load compared to CB, and F with moderate to large effect sizes observed. However, despite not covering the greatest overall distance, FP (1264 m) covered the most distance at high speeds, compared to the 1145 m, 1004 m, and 798 m covered by CM, CB, and F, respectively. FP, CB, and CM averaged a similar number of sprints per game, ranging from 14 to 17 sprints, which were all notably higher than the 11 completed by F. While CB spent a greater percentage of their time walking and standing, they also spent a greater percentage sprinting compared to the other position groups. Previous research in elite men’s soccer has also reported CM position groups to cover the greatest distance during match play [7]. In analysis of 20 Spanish Premier League and 10 Champions League matches, Di Salvo et al. [7] reported that distances covered by central defenders were shorter than other position groups. Such differences at the elite level are likely a result of tactical and skill differences needed to perform at a higher level. For example, elite center defenders likely read the game flow better and anticipate potential outcomes more efficiently, ultimately requiring less overall running compared to defenders at the collegiate level. Additionally, at the elite level, there tends to be less kicking and running with more controlled passing, which may also require defenders to chase less and reduce overall sprinting throughout the match. Therefore, it is important to continue to examine differences in match demands across each level of play and between the men’s and women’s divisions.

Although this study presents novel findings on match demands of women’s collegiate soccer, it is not without limitations. First, the study was conducted in the upper Midwest region of the United States. Match demands may vary across various regions due to differences in environmental conditions. Next, the terminology used to describe position groups may not be applicable to all levels of competition or at the international level, making it challenging to draw direct comparisons across studies. Similarly, the use of different monitoring technologies, (i.e., video analysis, LPS, etc.) make it challenging to draw comparisons across the literature, as each system may provide propriety metrics to classify match demands or exhibit varying degrees of accuracy. There is also the potential for bias regarding the tactical strategies and style of match play of the team undergoing analysis.

## 5. Conclusions

The results of this study indicate a high degree of aerobic loading in Division III women’s soccer with HR values averaging 74% of HR max and reaching near max intensity at times with an average energy expenditure of 1300 kcals per match. This level of energy expenditure may warrant targeted nutritional interventions during periods of high match congestion to promote adequate recovery. Individualized training and recovery protocols may be warranted to accommodate differences in the physiological demands of each position group. CM, CB, and FP athletes may require higher training loads throughout the preseason period to elicit appropriate training adaptations in preparation for the upcoming season. Future research should examine what ratio of training load is optimal during the preseason and practice periods for adequately preparing the players for the physical demands of match play. Further, research should explore how match demands influence the time course of recovery throughout the season and effective recovery strategies. The match demands of NCAA Division III women’s soccer appear to be comparable with NCAA Division I soccer with the exception of a slightly higher high-speed distance in Division I match play compared to Division III. Additionally, the average velocity of NCAA Division I men match play appears to be faster than collegiate women. Compared to the elite level, NCAA Division III match demands appear to require less total and high-speed distances and a lower number of sprints, yielding a lower percentage of max HR than that reported in elite women. CM covered the most distance per match resulting in the highest training load. FP covered the most distance at high speeds, and the mean HR values were highest in the CM, CB, and FP positions, which resulted in the highest energy expenditures. Compared to other field-based intermittent team sports, Division III college soccer appears to cover greater total and high-speed distances with similar HR responses.

## Figures and Tables

**Table 1:** A summary of match demands across position groups.

Variable	GK	F	CM	CB	FP	All Players
**Distance (m)**	5622 ± 1953	7831 ± 2180	10,575 ± 511	9956 ± 2511	10,056 ± 2763	9793 ± 2715
**Training Load (AU)**	145 ± 66	185 ± 46	279 ± 69	226 ± 60	240 ± 79	240 ± 79
**Energy Expenditure (kcals)**	1167 ± 405	1038 ± 232	1324 ± 273	1349 ± 312	1232 ± 339	1275 ± 321
**Average HR (bpm)**	121 ± 29	133 ± 7.5	147 ± 9.9	144 ± 23	144 ± 21	142 ± 20
**Average HR (%)**	67.5 ± 7.5	70.2 ± 4.0	75.7 ± 5.7	73.6 ± 6.0	73.8 ± 5.3	73.7 ± 6.2
**Number of Sprints (n)**	4.5 ± 3.4	11.4 ± 4.6	16.6 ± 7.9	15.9 ± 6.9	17.7 ± 7.4	15.3 ± 8.0
**Low Accelerations (n)**	900 ± 323	763 ± 183	991 ± 250	996 ± 232	919 ± 264	953 ± 260
**Moderate Accelerations (n)**	26.7 ± 11.3	50.5 ± 14.2	69.7 ± 21.6	67.2 ± 18.7	68.9 ± 20.6	63.7 ± 23.3
**High Accelerations (n)**	2.8 ± 2.8	7.1 ± 3.7	10.9 ± 6.0	10.4 ± 5.3	11.7 ± 5.3	10.0 ± 5.9
**Low Decelerations (n)**	1006 ± 343	820 ± 190	1038 ± 252	1057 ± 236	970 ± 274	1010 ± 266
**Moderate Decelerations (n)**	22.9 ± 10.1	54.8 ± 15.0	76.9 ± 23.9	71.9 ± 19.1	73.9 ± 24.4	68.5 ± 26.7
**High Decelerations (n)**	3.5 ± 2.8	10.2 ± 5.0	11.6 ± 5.6	14.2 ± 5.5	16.5 ± 8.4	16.5 ± 8.4
**Walk/Stand Distance (m)**	4537 ± 1565	3176 ± 786	4138 ± 831	4673 ± 1242	4310 ± 1261	4299 ± 1182
**Jog Distance (m)**	1055 ± 490	3857 ± 1289	5420 ± 1349	4207 ± 1202	4471 ± 1342	4358 ± 1744
**Run Distance (m)**	41.5 ± 23.5	658 ± 253	916 ± 276	685 ± 306	836 ± 371	739 ± 389
**Sprint Distance (m)**	6.7 ± 14.5	140 ± 65.3	266 ± 117	309 ± 163	403 ± 258	282 ± 205
**HSD (m)**	48 ± 31	798 ± 308	1145 ± 388	1004 ± 417	1264 ± 613	1019 ± 560

Data are mean ± SD; Acceleration/Deceleration thresholds: Low = ±0.5–1.99 m·s^−2^, Moderate = ±2.00–2.99 m·s^−2^, High = ±3.00–50.0 m·s^−2^; Speed zone thresholds: Walk/Stand = <6.99 km·h^−1^, Jog = 7.0–14.99 km·h^−1^, Run = 15.0–18.99 km·h^−1^, Sprint = >19.00 km·h^−1^; HSD = >15 km·h^−1^. GK = goal keepers; CB = center defenders; FP = flank players; F = forwards; and CM = center midfielders; HR = heart rate; HSD = high-speed distance.

**Table 2:** Mean differences in external and internal load and movement characteristics between position groups, mean difference (MD) ± 95% confidence intervals and effect sizes.

Variable	F-CM	CM-CB	CB-FP	FP-CM	F-FP	CB-F
Distance (m)	−2744 (−4,275, −1213) * ES: −1.17 (M)	619 (−229, 1467) ES: −0.25 (S)	−100 (−989, 789) * ES: −0.04 (T)	518 (−1321, 283) * ES: −0.20 (S)	−2225 (−3779, −672) ES: −0.89 (M)	2124 (547, 3702) * ES: 0.90 (M)
Training Load (AU)	−94.7 (136.9, −52.5) * ES: −1.60 (L)	55.8 (32.4, 79.2) * ES: 0.82 (M)	−22.8 (−47.3, 1.7) ES: −0.20 (S)	−33.0 (−55.1, −10.9) * ES: −0.53 (S)	−61.7 (−104.5, −18.8) * ES: −0.85 (M)	38.9 (−4.6, 82.4) * ES: 0.77 (M)
Energy Expenditure (kcal)	−287 (−481, −91.8) * ES: −1.13 (M)	−10.3 (−118, 97.7) ES: −0.09 (T)	96.7 (−16.5, 209) * ES: 0.36 (S)	−86.4 (−188, 15.8)* ES: −0.29 (S)	−200 (−398, −2.5) * ES: −0.67 (S)	297 (96.2, 497) * ES: 1.13 (M)
Average HR (bpm)	−18.2 (−25.9, −10.5) * ES: −1.59 (L)	7.5 (0.4, 14.6) * ES: 0.17 (T)	6.1 (−0.5, 12.8) ES: 0.0 (T)	−13.6 (−19.3, −7.8) * ES: −0.18 (T)	−4.6 (−11.9, 2.7) ES: −0.70 (M)	10.7 (2.3, 19.1) * ES: 0.64 (S)
Average HR (%)	−5.5 (−11.4, 0.4) * ES: −1.11 (M)	3.2 (−0.1, 6.5) * ES: 0.36 (S)	−0.6 (−4.0, 2.8) ES: −0.04 (T)	−2.6 (−5.7, 0.5) * ES: −0.34 (S)	−2.8 (−8.9, 3.1) ES: −0.77 (M)	2.2 (−3.8, 8.4) ES: 0.67 (S)
Number of Sprints (n)	−4.8 (−6.8, −2.8) * ES: −0.80 (M)	1.0 (−0.9, 2.8) ES: 0.09 (T)	0.49 (−1.2, 2.2) ES: −0.25 (S)	−1.5 (−2.9, 0.01) ES: 0.14 (T)	3.3 (−5.2, −1.4) * ES: −1.02 (M)	3.8 (1.6, 5.9) * ES: 0.77 (M)
HSD (m)	−318 (−459, −177) * ES: −0.99 (M)	58.9 (−71.6, 189) ES: 0.35 (S)	77.9 (−44.1, 199) ES: −0.50 (S)	−137 (−241, −32.3) * ES: 0.23 (S)	−180 (−314, −47.5) * ES: −0.96 (M)	259 (104, 414) * ES: 0.56 (S)
Low Accelerations (n)	−210 (−306, −116) * ES: −1.04 (M)	25.1 (−62.7, 113) ES: −0.02 (T)	151 (68.5, 233) * ES: 0.31 (S)	−176 (−246, −106) * ES: −0.28 (S)	−35.2 (−125, 54.4) ES: −0.69 (M)	186 (81.7, 289) * ES: 1.12 (M)
Moderate Accelerations (n)	−19.5 (−26.8, −12.1) * ES: −1.05 (M)	2.5 (−4.3, 9.3) ES: 0.12 (T)	9.2 (2.9, 15.6) * ES: −0.09 (T)	−11.7 (−17.1, −6.3) * ES: −0.04 (T)	−7.7 (−14.5, −0.8) * ES: −1.04 (M)	16.9 (8.9, 25.0) * ES: 1.06 (M)
High Accelerations (n)	−3.5 (−4.9, −2.0) * ES: −0.76 (M)	0.6 (−0.7, 1.9) ES: 0.09 (T)	0.1 (−1.1, 1.4) ES: −0.25 (S)	−0.7 (−1.8, 0.3) ES: 0.14 (T)	−2.7 (−4.1, −1.4) * ES: −1.01 (M)	2.9 (1.3, 4.5) * ES: 0.72 (M)
Low Decelerations (n)	−201 (105, 299) * ES: −0.98 (M)	26.1 (−63.6, 116) ES: −0.08 (T)	145 (61.3, 229) * ES: 0.34 (S)	−171 (−243, −99.6) * ES: −0.26 (S)	−30.2 (−122, 61.3) ES: −0.63 (S)	175 (69.1, 282) * ES: 1.11 (M)
Moderation Decelerations (n)	−19.3 (−27.2, −11.3) * ES: −1.1 (M)	2.9 (−4.3, 10.3) ES: 0.23 (S)	11.4 (4.5, 18.2) * ES: −0.09 (T)	−14.4 (−20.2, −8.5) * ES: −0.12 (T)	−4.9 (−12.4, 2.6) ES: −0.82 (M)	16.3 (7.6, 24.9) * ES: 0.99 (M)
High Decelerations (n)	−1.8 (−3.6, 0.1) ES: −0.26 (S)	−1.6 (−3.3, 0.2) ES: −0.47 (S)	0.4 (−1.2, 2.0) ES: −0.32 (S)	1.1 (−0.2, 2.5) ES: 0.69 (M)	−2.9 (−4.6, −1.1) * ES: −0.91 (M)	3.3 (1.3, 5.4) * ES: 0.76 (M)
Walk/Stand Distance (m)	−839 (−1685, 7.4) * ES: −1.19 (M)	−535 (−1,095, 24.5) ES: −0.51 (S)	363 (−221, 947) ES: 0.29 (S)	172 (−358, 702) ES: 0.16 (T)	−1134 (−2123, −145) * ES: −1.08 (M)	1497 (491, 2503) * ES: 1.44 (L)
Jog Distance (m)	−1563 (−2630, −496) * ES: −1.19 (M)	1213 (600, 1825) * ES: 0.95 (M)	−264 (−903, 374) ES: −0.21 (S)	−949 (−1529, −369) * ES: −0.71 (M)	−615 (−1697, 468) ES: −0.47 (S)	350 (−749, 1451) ES: 0.28 (S)
Run Distance (m)	−257 (−513, −2.8) * ES: −0.97 (M)	230 (83.9, 377) * ES: 0.79 (M)	−150 (−303, 2.5) ES: −0.44 (S)	−80.1 (−218, 58.6) ES: −0.24 (S)	−178 (−437, 80.9) ES: −0.56 (S)	27.5 (−235, 290) ES: 0.09 (T)
Sprint Distance (m)	−126 (−273, 20.8) ES: −1.33 (L)	−43.2 (−128, 41.1) ES: −0.30 (S)	−93.9 (−182, −5.9) * ES: −0.44 (S)	137 (57, 217) * ES: 0.68 (M)	−263 (−412, −114) * ES: −1.40 (L)	169 (17.9, 321) * ES: 1.36 (L)

Acceleration/Deceleration thresholds: Low = ±0.5–1.99 m·s^−2^, Moderate = ±2.00–2.99 m·s^−2^, High = ±3.00–50.0 m·s^−2^; Speed zone thresholds: Walk/Stand = <6.99 km·h^−1^, Jog = 7.0–14.99 km·h^−1^, Run = 15.0–18.99 km·h^−1^, Sprint = >19.00 km·h^−1^; HSD = >15 km·h^−1^. GK = goal keepers; CB = center defenders; FP = flank players; F = forwards; and CM = center midfielders; HR = heart rate; HSD = high-speed distance; T = Trivial; SM = Small; M = Moderate; L = Large; VL = Very large for effect size classification. * Denotes significant difference (*p* < 0.05).
